# Comparative study of early versus interval appendicectomy in appendicular mass

**DOI:** 10.6026/973206300215011

**Published:** 2025-12-15

**Authors:** Rakesh Shakya, Sandeep Sharma, Kaushlendra Singh Narwariya, Krishna Pratap Singh, Parul Nema

**Affiliations:** 1 Department of General Surgery, SRVS Medical College Shivpuri, Madhya Pradesh, India; 2 Department of General Surgery, Motilal Nehru Medical College Prayagraj, Uttar Pradesh, India; 3 Department of Pathology, SRVS Medical College Shivpuri, Madhya Pradesh, India

**Keywords:** Appendicular mass, early appendectomy, early appendectomy, postoperative outcomes, comparative study

## Abstract

The optimal timing for appendectomy in patients with appendicular mass remains debated, with early versus interval appendectomy being two common approaches. Therefore, it is of interest to compare clinical outcomes of early appendectomy and interval appendectomy in 184 patients presenting with appendicular mass. A prospective observational design divided patients equally into early surgery within 24-48 hours and interval surgery after 6-8 weeks of conservative management. Early appendectomy demonstrated shorter operative time and faster postoperative recovery. Hospital stay and return of bowel function were significantly improved in the early appendectomy group, with similar complication rates between groups. Overall, early appendectomy proved to be a safe and efficient option for managing appendicular mass. Early appendectomy for appendicular mass results in shorter operative time, faster postoperative recovery and improve hospital stay and bowel function, proving it to be a safe and efficient option compared to interval appendectomy.

## Background:

Appendicular mass, a localized inflammatory mass resulting from acute appendicitis, is a common clinical presentation in both pediatric and adult populations. The management of appendicular mass remains a subject of debate, particularly concerning the optimal timing for surgical intervention. Traditionally, conservative management with antibiotics and observation has been employed, followed by interval appendectomy after 6-8 weeks. This approach aims to reduce the risk of intraoperative complications by allowing the inflammatory process to subside [1]. However, recent studies have questioned the necessity of delaying surgery. Early appendectomy, defined as surgical intervention within 24-48 hours of diagnosis, has been proposed as an alternative to the traditional interval approach. Proponents argue that early surgery may reduce the total length of hospital stay and time away from normal activities [2, 3]. Additionally, early appendectomy may decrease the incidence of abdominal abscess formation, a common complication in patients with appendicular mass [4, 5]. Despite these potential benefits, the evidence comparing early and interval appendectomy is limited and of low certainty. A Cochrane review found that early appendectomy may reduce the rate of abdominal abscesses and shorten hospital stays; however, the evidence was very uncertain and further trials are needed to confirm these findings [6]. Therefore, it is of interest to compare the clinical outcomes of early versus interval appendectomy in patients presenting with appendicular mass, focusing on operative findings, postoperative complications, length of hospital stay and time to recovery.

## Methodology:

This prospective observational study was conducted at an Indian teaching hospital. Informed written consent was obtained from all participants after explaining the study protocol and potential risks and benefits. A total of 184 patients diagnosed with appendicular mass based on clinical evaluation and corroborative imaging studies, including ultrasonography or computed tomography of the abdomen, were included in the study. Diagnosis was established using a combination of history, physical examination, laboratory investigations and radiological findings. Patients were categorized into two groups: the early appendectomy group, in which surgery was performed within 24- 48 hours of presentation and the interval appendectomy group, in which patients were initially managed conservatively and underwent appendectomy after a period of 6-8 weeks. Exclusion criteria included patients with generalized peritonitis, appendicular abscess requiring immediate drainage, prior abdominal surgery, pregnancy, or comorbid conditions that contraindicated anesthesia or surgery. Patients with recurrent appendicular mass or those unwilling to participate were also excluded from the study. All participants underwent standardized preoperative evaluation including complete blood counts, serum electrolytes, renal and liver function tests and coagulation profile.

Imaging studies were performed to assess the extent of the appendicular mass, rule out abscess formation and guide management planning. Conservative management of patients in the interval appendectomy group consisted of intravenous fluids, broad-spectrum antibiotics tailored to local antibiogram, analgesics and bowel rest. Patients were closely monitored with daily clinical assessments, including vital signs, abdominal examination and laboratory investigations to detect any signs of clinical deterioration. Surgical intervention was performed under general anesthesia using either an open or laparoscopic approach, depending on surgeon discretion and intraoperative findings. Intraoperative findings including the condition of the appendix, presence of adhesions, perforation, or abscess formation were carefully documented. Postoperative management included administration of intravenous antibiotics, analgesics, early mobilization, monitoring for complications such as wound infection, intra-abdominal abscess, ileus, or bleeding and gradual reintroduction of oral diet as tolerated. Length of hospital stay, time to return of bowel function and postoperative complications were recorded. Data collection was performed using a structured proforma and all information was entered into a secure database for analysis. Statistical analysis was conducted using SPSS version 26.0, with continuous variables expressed as mean ± standard deviation and categorical variables expressed as frequencies and percentages. Comparison between groups was performed using Student's t-test for continuous variables and chi-square test or Fisher's exact test for categorical variables. A p-value <0.05 was considered statistically significant.

## Results:

A total of 184 patients with appendicular mass were included in the study, with 92 patients each in the early appendectomy (EA) and interval appendectomy (IA) groups. Demographic and baseline clinical characteristics, including age, sex distribution, duration of symptoms, presence of fever, abdominal tenderness and leukocyte counts, are summarized in Table 1 (see PDF). Operative findings, such as adhesions, perforation, abscess formation, conversion to open surgery and operative time, are presented in Table 2 (see PDF). Postoperative outcomes, including wound infection, intra-abdominal abscess, ileus, length of hospital stay, time to return of bowel function and readmission rates, are shown in Table 3 (see PDF). Comparative analysis indicates that early appendectomy was associated with shorter operative time, reduced length of hospital stay and faster recovery of bowel function, while the rates of postoperative complications were comparable between the two groups.

## Discussion:

This study aimed to compare the clinical outcomes of early appendectomy (EA) and interval appendectomy (IA) in patients presenting with appendicular mass. Our findings suggest that both approaches are effective; however, IA was associated with a lower incidence of postoperative complications and a shorter operative duration. Previous studies have highlighted the benefits of IA in reducing postoperative complications. For instance, Suzuki *et al.* reported that IA after conservative treatment for appendicitis with abscess was associated with fewer complications compared to EA. They found that the rates of ileocecal resection and postoperative complications were significantly lower in the IA group than in the EA group (3% vs. 50%, P < 0.001 and 9% vs. 75%, P < 0.001, respectively) [7]. Similarly, a retrospective study by Tarar *et al.* demonstrated that IA was a safe and feasible treatment approach after conservative treatment for appendicitis with abscess, with no significant increase in complications [8]. In contrast, early surgical intervention, while effective, may be associated with higher complication rates. A Cochrane review by Zhou *et al.* indicated that early appendectomy may reduce the total length of hospital stay and increase the time away from normal activities, but the evidence is very uncertain regarding its impact on overall morbidity and other complications [6]. Furthermore, the risk of incidental appendiceal neoplasms is a concern in patients undergoing appendectomy. Studies have shown that the rate of unexpected malignancy increases with age, reaching a peak of 16% in patients under the age of 50 [9]. This underscores the importance of considering IA, which allows for preoperative evaluation and planning. This study offers several strengths, including its prospective design, which enhances the reliability of the findings by minimizing recall bias. The inclusion of a relatively large sample size of 184 patients allows for a more robust comparison between early and interval appendectomy approaches. Additionally, the study was conducted at a single tertiary care center, ensuring uniformity in surgical techniques and postoperative care protocols. The comprehensive data collection on operative time, postoperative complications and length of hospital stay provides valuable insights into the comparative effectiveness of the two surgical strategies [10]. However, the study also has limitations. Being a single-center study, the results may have limited generalizability to other settings with different patient populations or healthcare infrastructures. The absence of randomization introduces potential selection bias, as patient allocation to either early or interval appendectomy was not randomized. Furthermore, the study did not account for long-term outcomes, such as recurrence rates or quality of life post-surgery, which are crucial for evaluating the long-term efficacy of the surgical approaches. Additionally, the study did not assess the impact of laparoscopic versus open surgical techniques, which could influence postoperative recovery and complication rates. Lastly, the lack of a control group receiving conservative management without surgery limits the ability to draw definitive conclusions about the superiority of early or interval appendectomy over non-surgical approaches [11].

## Conclusion:

Early appendectomy in patients with appendicular mass is a safe and effective approach, providing the advantages of shorter operative time, reduced hospital stay and faster recovery of bowel function compared to interval appendectomy. Both strategies demonstrate comparable rates of postoperative complications, indicating that patient selection and clinical judgment remain crucial in guiding management. Thus, early surgical intervention may offer improved efficiency and patient outcomes in appropriately selected cases of appendicular mass.
